# Mathematical Modeling of Therapy-induced Cancer Drug Resistance: Connecting Cancer Mechanisms to Population Survival Rates

**DOI:** 10.1038/srep22498

**Published:** 2016-03-01

**Authors:** Xiaoqiang Sun, Jiguang Bao, Yongzhao Shao

**Affiliations:** 1 Zhong-shan School of Medicine, Sun Yat-Sen University, Guangzhou 510089, China; 2 School of Mathematical and Computational Science, Sun Yat-Sen University, Guangzhou 510000, China; 3 School of Mathematical Sciences, Beijing Normal University, Beijing 100875, China; 4 Department of Population Health, NYU School of Medicine, New York University, New York, NY 10016, USA; 5 Interdisciplinary Melanoma Collaborative Group, NYU Cancer Institute, New York, NY 10016, USA

## Abstract

Drug resistance significantly limits the long-term effectiveness of targeted therapeutics for cancer patients. Recent experimental studies have demonstrated that cancer cell heterogeneity and microenvironment adaptations to targeted therapy play important roles in promoting the rapid acquisition of drug resistance and in increasing cancer metastasis. The systematic development of effective therapeutics to overcome drug resistance mechanisms poses a major challenge. In this study, we used a modeling approach to connect cellular mechanisms underlying cancer drug resistance to population-level patient survival. To predict progression-free survival in cancer patients with metastatic melanoma, we developed a set of stochastic differential equations to describe the dynamics of heterogeneous cell populations while taking into account micro-environment adaptations. Clinical data on survival and circulating tumor cell DNA (ctDNA) concentrations were used to confirm the effectiveness of our model. Moreover, our model predicted distinct patterns of dose-dependent synergy when evaluating a combination of BRAF and MEK inhibitors versus a combination of BRAF and PI3K inhibitors. These predictions were consistent with the findings in previously reported studies. The impact of the drug metabolism rate on patient survival was also discussed. The proposed model might facilitate the quantitative evaluation and optimization of combination therapeutics and cancer clinical trial design.

Drug resistance places an often inevitable limit on the long-term effectiveness of targeted therapeutics for cancer patients^1^,^2^. Considerable efforts have been made to combat drug resistance and improve patient survival. Although the underlying molecular and cellular mechanisms are complex, some paradigms of drug resistance mechanisms have been established^3^,^4^,^5^,^6^,^7^,^8^.

It is widely acknowledged that the inherent heterogeneity^9^,^10^ of cancer cell populations, which is assumed containing both drug-sensitive and drug-resistant cells, contributes to drug resistance and metastasis^11^,^12^,^13^,^14^. A recent study^15^ revealed a novel drug resistance mechanism in which drug-sensitive cancer cells secrete various soluble factors (e.g., IGF and HGF) into the tumor microenvironment in response to targeted therapy. These secreted factors can promote the growth, dissemination and metastasis of drug-resistant cancer cells and support the survival of drug-sensitive cells. Therefore, microenvironment adaptation^16^ plays an important role in the rapid emergence of acquired drug resistance.

Evaluating cancer therapeutics in the context of tumor heterogeneity and microenvironment adaptation is very complex. In traditional *in vitro* and *in vivo* experiments, multiple cell types and multiple drug dosages must be considered, in addition to other experimental conditions and challenges in human population studies. As such, these studies are expensive and time consuming. Therefore the systematic development of effective therapeutics to overcome drug-resistance mechanisms has posed a major challenge. Mathematical modeling may potentially serve to bridge molecular/cellular mechanisms of drug resistance and population-level patient survival, and facilitates the quantitative evaluation and optimization of combination therapeutics and cancer clinical trial design.

Many mathematical and computational models have been developed to simulate tumor growth and drug response. For example, the cellular automata model^17^,^18^ or agent-based model^19^,^20^,^21^, continuum partial differential equations model^22^,^23^ and hybrid discrete-continuum model^24^,^25^ have all been applied to evaluate tumor growth at the molecular, cellular and/or tissue level. These models have substantially advanced our understanding of tumor initiation and progression. However, due to their complexity and/or intensive computing burden, these models have rarely been applied to predict population-scale patient survival. Haeno *et al.*^26^ developed a mathematical framework to describe pancreatic metastasis using a branching process to help understand cancer growth dynamics during metastasis and identify optimal therapeutic interventions. However, this framework focused on genetic mutation-induced drug resistance and did not address the role of targeted therapy-induced microenvironment adaptations in drug resistance. The use of combination therapy has been suggested in cases of drug resistance, such as in advanced melanoma patients with BRAF mutations^15^,^16^. Therefore, the development of mathematical models capable of quantitatively evaluating synergism in combination drug therapy is desirable.

In this study, we created a multiscale model comprising a set of stochastic differential equations driven by both the Wiener process and Poisson process to describe pharmacokinetics, cellular dynamics, and progression-free survival at the patient level while accounting for microenvironment adaptations (Fig. 1). Our model was subsequently verified using population- and cellular-scale clinical data. Then, we evaluated the efficiency and synergy of different combination therapies (combinations of BRAF, MEK and PI3K inhibitors). Our modeling revealed that different patterns of synergy existed for these combinations. Finally, sensitivity analysis revealed several key parameters that may combine with each other to affect the cancer cellular dynamics and patient survival, and facilitates the quantitative evaluation and design of combination therapeutics. In addition, we examined the impacts of different treatment schedules and drug metabolism rates on patient survival.

## Models

### Cellular dynamics

Tumors are heterogeneous (e.g., in their mutation profiles), resulting in some tumor cells possessing sensitivity to drug therapy and others in the same tumor exhibiting resistance to it. To model growth, transition and dissemination dynamics in drug-sensitive and drug-resistant cancer cells in patients with metastatic disease, we employed the following set of stochastic differential equations (SDEs):


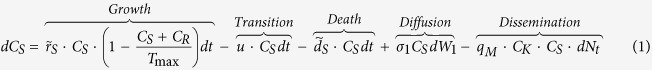



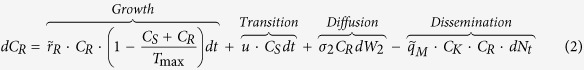


where 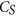 and 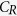 represent relative numbers (assumed in the unit of 10^8^ 26,27,28) of drug-sensitive cancer cells and drug-resistant cancer cells, respectively. The first terms on the right-hand side of equation (1) and equation (2) describe the growth of sensitive cells and resistant cells, respectively. 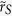 and 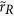 are growth rate coefficients associated with these two cell types. The growth of drug-sensitive and drug-resistant tumor cells was assumed to follow a logistic growth law^29^,^30^. 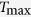 represents maximal carrying capacity. The second terms in equation (1–2) describe the transition from sensitive cells to resistant cells, e.g. due to genetic/epigenetic mutations. *u* represents the mutation rate in drug-sensitive cells as they convert to drug-resistant cells (i.e., mutation-driven drug resistance). The third term in equation (1) describes the drug-induced death of drug-sensitive cells. 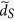 is the death rate of drug-sensitive tumor cells following treatment (e.g., BRAF inhibitors for V600 mutated melanoma) and depends on drug concentration (*D*) via the equation 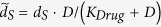, where 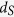 represents maximal death rate, and 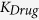 is a Michaelis constant representing the drug concentration associated with reaching the half-maximal inhibition effect. The fourth term (also called a diffusion term) in equation (1) simulates the stochastic fluctuation of cell numbers and is modeled by the standard Wiener process *W* that is described as 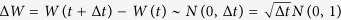, where *N*(0,1) is a unit normal distribution. The third term in Equation (2) is similar. 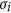 (*i* = 1, 2) represents the diffusion rate. The last terms in equation (1–2) describe the dissemination of existing cancer cells.

Independently of 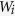, 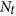 represents a Poisson process with intensity *λ* and describes the count of metastasis within a cancer cell population^31^,^32^. Specifically, the Poisson process 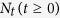 is characterized by 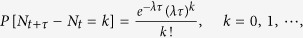 where *λ* is the expectation of disseminating cell number within per unit time (Day). In addition, 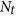 has independent increments, and 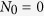. In the above equations (1–2), both drug-sensitive and drug-resistant cancer cells were assumed to have the potential to further metastasize. 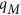 and 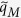 represent the dissemination rates of drug-sensitive and drug-resistant cells, respectively. 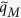 is regulated by drug-induced resistance factors as described below. It should be noted that the metastasized cells in patients before therapy were considered to be included in these sensitive or resistant cells, and a new variable was introduced to account for new metastasis after the initiation of targeted therapy as follows.

Therapy-induced drug resistance can intensify tumor metastasis^15^,^16^. The growth of new metastatic tumor cells following the drug treatment was modeled using a SDE driven by a jump process as follows:


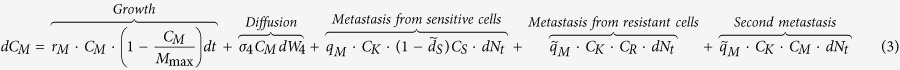


where 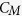 represents the number of new metastatic cells after the initiation of new therapy. The first term in equation (3) describes the growth of the metastatic cells, and 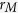 is a metastatic cell growth rate coefficient. 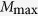 is the maximal carrying capacity of metastatic cell growth. The second term (diffusion term) simulates fluctuation of metastatic cell population as mentioned above. Metastasis from existing cancer and metastatic emissions by the metastases themselves (i.e., secondary metastasis)^33^ were taken into account, which were modeled in the last three terms of equation (3). 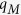 and 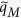 respectively represent dissemination rates of drug-sensitive and drug-resistant cancer cells as described above. Metastatic rates were assumed to depend on existing tumor size (i.e. drug-sensitive/resistant cancer cell numbers 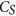 and 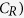
34 and angiogenic cell number 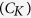
29. The drug effect on drug-sensitive metastatic cells was incorporated by using 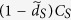 in the third term. By assuming that newly developed metastasis sites are more supportive of the growth of invasive cancer cells, a positive net increase rate of new metastatic cells due to the secondary metastasis was introduced in the last term of the above equation.

Angiogenic growth in tumors is induced by the secretion of angiogenic growth factors (e.g., VEGF). We modeled angiogenesis based on previously established work^35^ with the following equation:


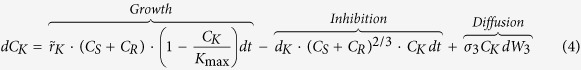


where 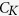 represents the number of angiogenic cells. The first term describes the growth of angiogenic cells induced by tumor cells. 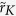 is a growth rate coefficient associated with angiogenic cells, and 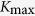 is the maximal carrying capacity for blood vessel growth. The second term describes the growth inhibition of angiogenic cells by tumor cells with a coefficient 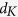. Newly grown blood vessels can provide tumor cells with nutrients (such as oxygen and glucose) and thus influence the maximal carrying capacity of tumor cells^29^,^30^ as follows: 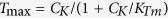, where 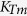 is a Michaelis constant.

### Pharmacokinetics

Pharmacokinetics describe the dynamics of drug absorption, metabolism and elimination by the body^36^. These processes are often modeled as follows^37^,^38^:


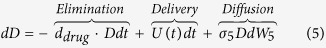


where *D* represents drug concentration in the body. The first term in the above equation models the first-order elimination rate of drugs, and 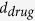 is a metabolic rate coefficient of patients. 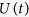 in the second term is the rate of drug delivery. Brownian motion was also assumed in the above equation to accommodate stochasticity^37^,^38^.

The initial conditions of the equations (1–4) were set to *C*_*S*_ = 0.2, *C*_*R*_ = 0.001 and *C*_*K*_ = 0.1, simulating the relative cell number (in the unit of 10^8^ 26,27,28) in patients (e.g. patients with metastatic melanoma and BRAF V600 mutations) before initiation of the new therapy. Starting from the initiation of the drug treatment, the number of new metastatic cells was counted, with a initial value *C*_*M*_ = 0. The initial concentration of drug was set to 0. The uniqueness of the solution to the above SDEs (equations (1–5)) were easily obtained, since their coefficients satisfies the appropriate growth conditions and are locally Lipschitz continuous^39^. We employed a time-adapted Euler scheme^40^ to provide numerical solutions to the SDEs driven by both diffusion (Brownian motion) and jump (Poisson process).

### Microenvironment adaptations to drug treatment

As demonstrated by recent experimental and preclinical studies^15^, when BRAF inhibitors (BRAF-I), such as Vemurafenib and Dabrafenib, are administered to cancer patients with BRAF mutations, they can induce drug-sensitive cancer cells to secrete resistance factors (e.g., IGF and HGF) into the tumor microenvironment. We modeled the secretion of drug-induced resistance factors (DIRFs) by drug-sensitive tumor cells according to Michaelis–Menten kinetics^41^ as follows:


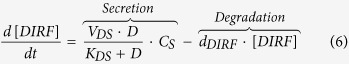


where 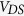 is the maximal secretion rate of DIRFs from drug-sensitive cells, and 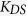 is a Michaelis constant representing the drug concentration at which a half-maximal secretion rate is achieved. 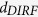 represents DIRF degradation rate.

It was assumed that DIRF secretion and/or degradation in the microenvironment are much faster processes than cellular phenotype switching and shifts in cellular population dynamics. Therefore, using a quasi-steady state assumption we can express the secreted DIRF concentration as follows:


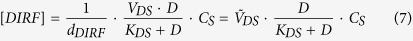


where 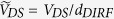.

Secreted DIRFs can promote outgrowth, dissemination and metastasis in drug-resistant cells and enhance survival in drug-sensitive cells^15^,^16^. The effects of DIRFs on cellular dynamics were modeled using the following functions, which correlate DIRF concentration to the growth, dissemination and metastasis rates of three types of cancer cells:


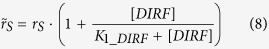



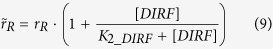



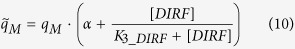



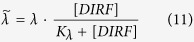


equation (8) describes the dependence of growth rate of sensitive cells on the secreted DIRF concentration, where 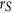 is a basal growth rate of sensitive cells, and 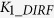 is a Michaelis constant of DIRF for regulating 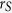. Equation (9) depicts the growth rate of resistant cells depending on the secreted DIRF concentration, where 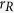 is the basal growth rate of resistant cells, and 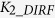 is a Michaelis constant of DIRF for regulating 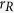. Equation (10) correlates DIRF concentration to the dissemination rate of cancer cells, where *α* is the regulatory coefficient, and 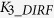 is a Michaelis constant of DIRF for increasing 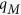. Equation (11) represents the metastasis rate regulated by DIRF concentration with 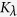 being a Michaelis constant.

In this way, we linked the short-term timescale (minutes) associated with intercellular signaling to the long-term timescale (days) necessary for cellular dynamics^42^,^43^,^44^.

### Progression-free survival analysis of a patient population

Cancer progression is often clinically evaluated using radiographic imaging. In the below analysis, if a patient’s total tumor cell number or tumor volume exceeded a pre-set threshold, 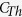 (assumed to be 1.6 in this work), then we considered the patient’s cancer to be progressing. Therefore, progression-free survival (PFS) time 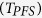 was defined as the length of time between initiation of therapy (t = 0, the starting time in our model) and initiation of cancer progression or death as follows:


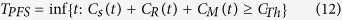


where 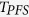 is a random variable due to the stochastic nature of cancer progression. In our simulations, *N*, which represents number of patients, was set to 100. We calculated the progression-free survival time for each patient in the simulation and then computed overall survival percentages and survival frequencies for the entire patient population under different treatment schedules. In the following text, patient survival refers to progression-free survival unless stated otherwise.

Most of the parameters used to assess cellular dynamics and pharmacokinetics were collected from previous studies^26^,^35^,^37^, while others, such as those representing microenvironment adaptations and metastasis, were calibrated using recent experimental^15^,^16^ and clinical data^45^. The calibration details are described in Supplementary Text S1 (See also Supplementary Fig. S1). The biological meaning underlying the parameters that were used and values of the parameters are listed in Supplementary Table S1. The sensitivity analysis (Supplementary Text S2) was discussed below, which demonstrated the robustness of the model predictions to the variations of less critical parameter values. The program was implemented using MATLAB R2007b (MathWorks).

### Incorporation of the effects of MEK and PI3K inhibitors into the model

Currently, in addition to BRAF inhibitors (e.g., Vemurafenib and Dabrafenib), several other targeted inhibitors, such as MEK inhibitors (e.g., Trametinib and Cobimetinib) and PI3K inhibitors (e.g., BEZ235) are in clinical trials for melanoma cancer patients. In this study, we investigated the synergy between BRAF inhibitors in conjunction with each of these other two inhibitor types by incorporating their effects into our model based on their different signaling mechanisms, respectively.

MEK is a downstream effector protein of RAF signaling^46^; therefore, we assumed that MEK inhibitors would produce similar effects to BRAF inhibitors when inducing the death of drug-sensitive cancer cells. It has been shown that both BRAF inhibitors and MEK inhibitors can increase the death rate of drug-sensitive cells. MEK inhibitors can also promote the secretion of drug-induced resistance factors (DIRFs) from drug-sensitive cells^15^ because RAF and MEK share the same downstream effector, transcription factor FRA1, which has been identified as a major regulatory factor of DIRF secretion. Therefore, the effects of MEK inhibitor use were incorporated into the model using Hill functions as in refs 47,48:


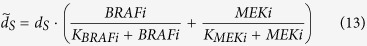



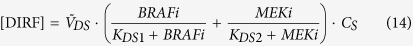


where 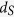 represents basal death rate. 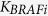 and 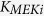 are Michaelis constants representing the BRAF inhibitor and MEK inhibitor concentrations at which half-maximal inhibition effects are reached. 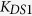 and 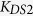 are also BRAF inhibitor and MEK inhibitor Michaelis constants for DIRF secretion. 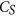 is the number of drug-sensitive cells.

In drug-resistant cells, the PI3-Kinase (PI3K)/AKT pathway is over-activated by DIRFs^15^; therefore, a PI3K inhibitor may repress DIRF-stimulated PI3K/AKT pathway activation and thus reduce DIRF effects. We used an inhibition Hill function^48^ to include the effects of PI3K inhibitor-mediated DIRF signal modification into our model:


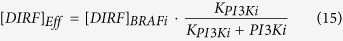


where 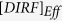 represents effective action of DIRF inhibited by PI3K inhibitor, and 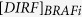 is the DIRF steady-state concentration following stimulation with a BRAF inhibitor. 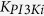 is the Michaelis constant for the PI3K inhibitor’s half-maximal inhibition concentration. In this simplified way, we incorporated the effects of PI3K inhibitor into the model without considering complex intracellular signaling networks. This strategy enabled us to reduce the complexity of the model.

It should be noted that we used dimensionless concentrations of BRAF inhibitor, MEK inhibitor and PI3K inhibitor in the simulation by respectively normalizing them to the Michaelis constants 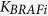,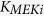 and 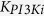, as in our previous study^48^. As such, we did not introduce any additional parameters into the model to further reduce the number of unknown parameters.

## Results

### *In silico* prediction of cellular kinetics and patient survival following drug treatment

**Figure 2:**
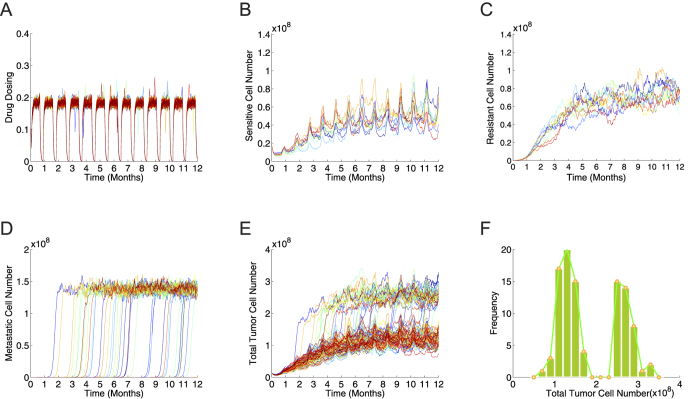
A typical simulation of cellular- and patient-level responses to drug treatment. A BRAF inhibitor was administered daily for 3 weeks, followed by 1 week off^45^. (**A**) Pharmacokinetics. Time courses of 100 samples showing numbers of (**B**) drug-sensitive cancer cells, (**C**) drug-resistant cancer cells, (**D**) new metastatic cells, and (**E**) total tumor cells. (**F**) Bimodal distribution of total tumor cell number at 360 days.

We investigated cellular response kinetics following drug treatment *in silico*. In Fig. 2, a typical simulation of BRAF-I treatment is shown. Figure 2A details BRAF-I kinetics for 100 cancer patients. The inhibitor was administered daily for 3 weeks followed by 1 week of no treatment, concordant with the drug schedule of a previous study^45^. Figure 2B,E show the time courses of all 100 samples with respect to numbers of drug-sensitive cancer cells, drug-resistant cancer cells, metastatic cells and total tumor cells. Drug-sensitive cancer cell growth was repressed following drug administration, but it periodically rebounded during no treatment weeks. Interestingly, metastatic cell growth (Fig. 2D) showed a similar pattern among the patients: an initial slow growth period (the length of which varied in different patients) followed by a rapid increase within ~1 month. The metastatic cell populations in different patients exhibited different transition times, resulting in heterogeneous sizes of cancer cells among patient population (Fig. 2E). Interestingly, this “all-or-no” metastasis causes a bimodal distribution for the number of total tumor cells after 12 months (Fig. 2F), indicating that cancer in some patients progressed but not yet in others.

For comparison, cellular dynamics without drug treatment are shown in Supplementary Fig. S2. In this case, the distribution (Supplementary Fig. S2D) of the number of total tumor cells was changed to an asymmetric bimodal distribution with decreased frequencies of cell numbers at high level (Supplementary Fig. S2E) due to the lack of the drug-induced metastasis (Supplementary Fig. S2C). We also applied our model to investigate the effects of targeted therapy on patient survival at the population level. Figure S2F shows the survival percentage of 100 cancer patients undergoing BRAF-I treatment from 0 to 360 days compared with no treatment controls. Our simulation demonstrated that treatment with BRAF-I significantly prolonged progression-free survival in melanoma patients harboring BRAF mutations. This result is consistent with clinical studies of melanoma patients harboring the BRAF V600E mutation^45^,^49^.

### Validation of cellular kinetics and patient survival using clinical data

**Figure 3:**
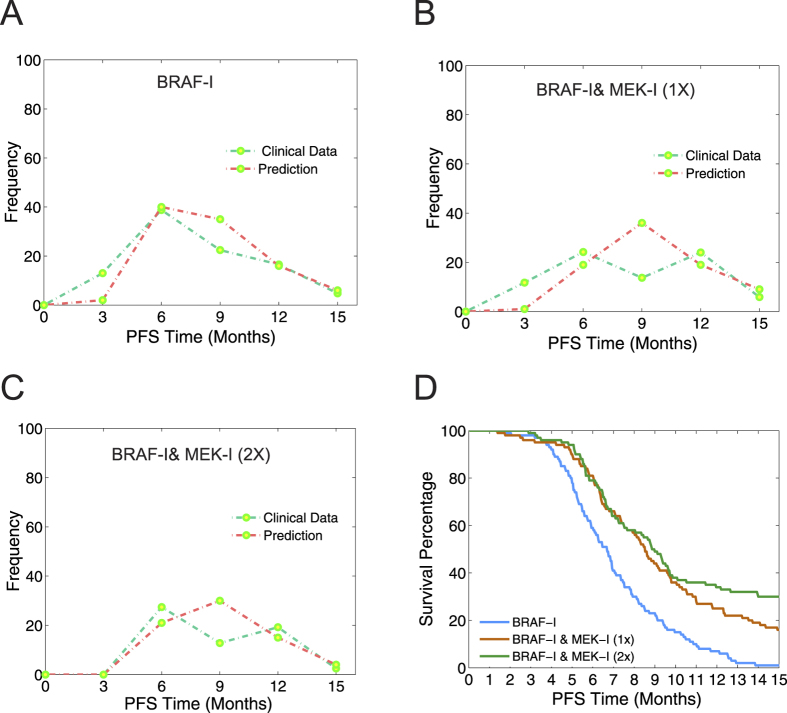
Validation of the model on the population scale by comparing survival frequency predictions with clinical survival data 50. We compared progression-free survival (PFS) time determined by the model prediction to that calculated from actual clinical data of a patient cohort administered Dabrafenib (a BRAF inhibitor) monotherapy (**A**), a combination of 150 mg Dabrafenib and 1 mg Trametinib (a MEK inhibitor) (BRAF-I&MEK-I, 1X) (**B**), and a combination of 150 mg Dabrafenib and 2 mg Trametinib (BRAF-I&MEK-I, 2X) (**C**). In the clinical data, there are 54 patients for each treatment group. In our simulation, 100 patients were simulated for each group. (**D**) The predicted progression-free survival percentages showed improved survival over time following the administration of combination therapeutics compared to BRAF-I monotherapy. The predicted survival curve shown here has a highly similar pattern to that in Fig. 1A of ref. 50.

We next validated our model using clinical data. In Fig. 3, a comparison of our model predictions with clinical population-scale survival data is shown^50^. The clinical data included distributions of progression-free survival times for 54 patients in each group treated either with Dabrafenib monotherapy (a BRAF-I) (Fig. 3A) or with a combination of BRAF-I and Trametinib (a MEK inhibitor, or MEK-I) at doses of 150 mg and 1 mg (1X; Fig. 3B) or 150 mg and 2 mg (2X; Fig. 3C). To simulate the conditions used to produce the clinical data, our model (red lines in Fig. 3A–C) examined the following three treatment strategies: BRAF-I alone, BRAF-I combined with 1 mg MEK-I, and BRAF-I combined with 2 mg MEK-I. It should be noted that the drug doses in the simulation have been normalized (refer to the Model section). We computed progression-free survival times using our model and compared them against the clinical data; the predicted and experimental results were in good agreement. Furthermore, as shown in Fig. 3D, our model predicted that the combination therapies enhanced progression-free survival more than the monotherapy, consistent with the clinical data.

**Figure 4:**
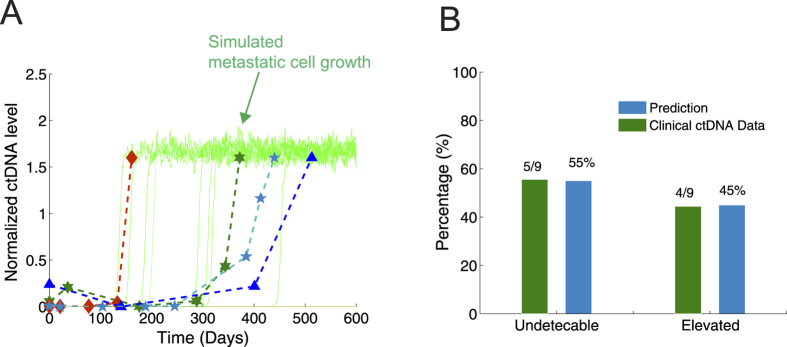
Validation of the model on the cellular scale by comparing metastatic cell growth patterns with normalized ctDNA data^52^. ctDNA concentrations were collected from 9 patients treated with BRAF and MEK inhibitors (BRAF-I and MEK-I) in combination. Increased ctDNA levels were evident in 4 of the 9 patients. These values were plotted against the predicted metastatic cell growth pattern for model validation. (**A**) Increased ctDNA concentrations in 4 patients co-treated with BRAF-I and MEK-I compared to simulated metastatic cell-growth curves. Both the ctDNA concentration data and the model prediction showed that some patients underwent an explosive growth pattern of metastatic cells. (**B**) Predicted distribution of the number of metastatic cells versus the clinical data.

Figure 4 shows a validation of the sudden increase observed in metastatic cell number in the model using clinical values of circulating tumor DNA (ctDNA). ctDNA has been proposed as a promising biomarker for monitoring metastatic cancers^51^. We used clinical data consisting of plasma ctDNA concentrations from 9 patients^52^ treated with BRAF-I and MEK-I in combination to verify the predicted metastatic cell growth. Of the 9 evaluated patients, 4 showed elevated ctDNA levels while undergoing combination therapy. In Fig. 4A, a comparison of the simulated growth pattern of metastatic cells with the clinical ctDNA data is shown. Both the clinical data and the model prediction showed a pattern of explosive metastatic cell growth in several patients. In addition, the model predicted that new metastatic cell numbers would either remain at an undetectable, low level (48%) or significantly increase (52%) (Fig. 4B). A threshold of metastatic cell number was set to 1, separating these two distinct levels. This prediction agrees with the clinical plasma ctDNA concentrations, in which ctDNA levels are either undetectable (5/9) or elevated (4/9).

### Evaluation of drug combination synergy

Currently, in addition to BRAF inhibitors (e.g., Vemurafenib and Dabrafenib), several other targeted inhibitors, including MEK inhibitors (e.g., Trametinib and Cobimetinib) and PI3K inhibitors (e.g., BEZ235), are being evaluated in clinical trials for treatment of melanoma patients. In the following study, we investigated whether the co-administration of BRAF-I with either MEK-I or PI3K inhibitor (PI3K-I) produces synergistic effects. To accomplish this, we incorporated the effects of these inhibitors into our model (see details in Model section) based on their specific signaling mechanisms.

A Bliss combination index^53^,^54^ was used to quantitatively evaluate the synergy produced by BRAF-I and MEK-I co-treatment and BRAF-I and PI3K-I co-treatment as follows:


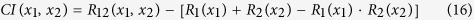


where 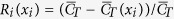 is the reduction of the median total tumor cell number 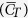 induced by the single BRAF inhibitor 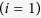, or either MEK inhibitor or PI3K inhibitor 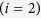 alone at dose 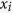.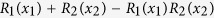 in equation (16) is the expected effect of combination therapy, and 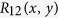 is the actual outcome produced by the combination. Hence, the combination of BRAF-I with MEK-I or PI3K-I is synergistic if *CI* > 0, antagonistic if *CI* < 0, and additive if *CI* = 0.

**Figure 5:**
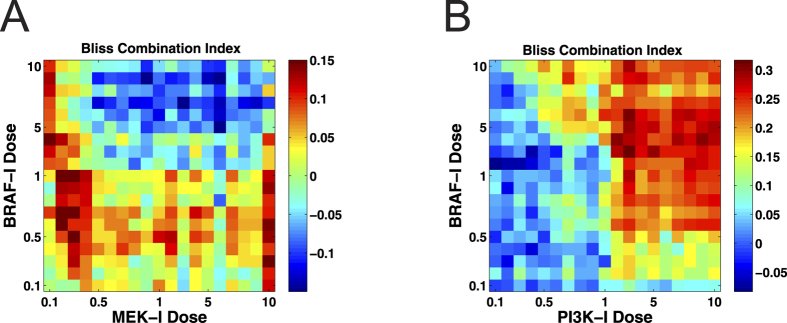
Evaluation of drug combinations for synergy using various concentrations of drugs. (**A**) Co-administration of BRAF and MEK inhibitors led to dose-dependent synergy. (**B**) Co-administration of BRAF and PI3K inhibitors led to dose-dependent synergy.

The model predicted that both the BRAF-I & MEK-I combination and the BRAF-I & PI3K-I combination possess dose-dependent synergy but in different patterns (Fig. 5). The BRAF-I & MEK-I combination was synergistic at lower combined dosages, while the BRAF-I & PI3K-I combination exhibited greater synergy at higher dosages.

Importantly, the predicted differences in the synergies of these two combinations are consistent with experimental studies^55^,^56^, which have reported stronger synergy for BRAF-I & PI3K-I co-treatment than BRAF-I & MEK-I co-treatment. Our model also predicted that the maximal CI value for the BRAF-I & PI3K-I combination (up to 0.3, Fig. 5A) is greater than that of the BRAF-I & MEK-I combination (less than 0.15, Fig. 5B). This demonstrates good agreement between our model’s predictions and the experimental data. In addition, the dose-dependent synergy predicted by our model might also help explain contradictory experimental observations that different PI3K/AKT inhibitors (e.g., MK2206 and Perifosine) produce opposing effects when combined with BRAF-I (PLX4032)^57^.

### Sensitivity analysis

**Figure 6:**
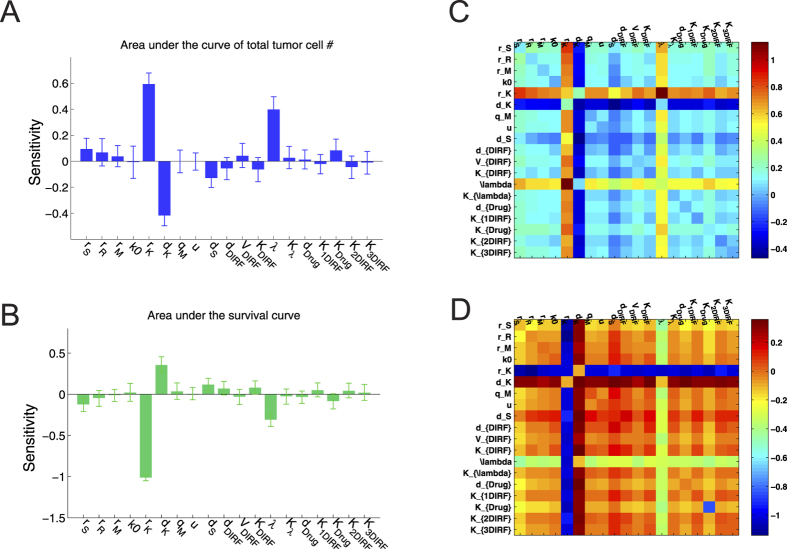
Sensitivity analysis. (**A**,**B**) Single-parameter sensitivity analysis. Sensitivity coefficients for (**A**) area under curve of total tumor cell number, and (**B**) area under the survival curve in response to variations in different parameter values. The computations were repeated 20 times, and the mean value and standard deviation of the sensitivity coefficients were calculated. The critical parameters involved in this model include *r*_*K*_, *d*_*K*_, and *λ*. (**C**,**D**) Two-parameter sensitivity analysis. The relative changes of the areas under the curves of (**C**) the total tumor cell number and (**D**) patient survival percentage with respect to the combinatorial variations in parameter values.

We conducted parameter sensitivity analysis (refer to Supplementary Text S2 for more details) to examine whether the model is robust to parameter variations and to identify parameters with critical effects on both cellular dynamics and patient survival. Figure 6A,B show a single-parameter sensitivity analysis. Here, a parameter was regarded to be sensitive/critical if its sensitivity coefficient is greater than 0.2. The results showed that the model was rather robust with respect to the variations of most parameters including those calibrated. Moreover, the following parameters were critical to model outputs: growth rate and death rate in angiogenic cells 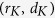 and metastatic rate (*λ*). Since angiogenesis positively supports the sustained growth of the tumor cells and provides an avenue for dissemination and translocation of metastatic cancer cells (especially drug-resistant metastatic cells), therefore the growth/death rates in angiogenic cells as well as metastatic rate of cancer cells were shown to significantly affect cellular dynamics and patient survival.

We further conducted a two-parameter sensitivity analysis to investigate how the parameters combine with each other to affect cellular dynamics and patient survival. The values of each pair of two different parameters were increased by 50% from their original values simultaneously. The computations were repeated 20 times, and the mean value of the sensitivity coefficients was calculated. Figure 6C,D show the relative changes of the areas under the curves of the total tumor cell number (Fig. 6C) and patient survival percentage (Fig. 6D) with respect to the combinatorial variations in parameter values. This global sensitivity analysis result revealed some interesting phenomena. For example, the combination of 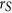 (growth rate of sensitive cells) and 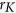, the combination of 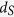 (drug-induced death rate of sensitive cells) and 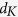, the combination of 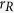 (growth rate of resistant cells) and 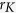, and the combination of 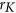 and *λ* show significant impacts on the tumor growth and patient survival. This also suggests that combining anti-angiogenic therapy with targeted therapy to combat drug resistance and cancer progression may improve cancer patient survival.

## Discussion

In this study, to examine therapy-induced drug resistance and cancer metastasis, we designed a novel stochastic model that connects the biological mechanisms underlying cancer drug resistance to population-level patient survival. A set of stochastic differential equations (SDEs) was used to model the dynamics of heterogeneous cellular populations containing drug-sensitive, drug-resistant, and metastatic cancer cells. An associated random variable characterizing progression-free survival time was subsequently defined. Our approach revealed several interesting features associated with cancer metastasis and progression kinetics. For example, dose-dependent synergy was evident in both BRAF-I and MEK-I co-treatment and BRAF-I and PI3K-I co-treatment. This result suggests that combination therapy with optimized dosages of inhibitors might reduce drug resistance.

Our model demonstrated that cancer metastasis and progression occur in bursts (Figs 2D,F and 4A). As such, metastasis may occur earlier than can be detected using common radiographic imaging approaches^58^. Furthermore, metastatic cancers may quickly progress after being detected. Based on these phenomena, new prognostic methods that offer much earlier detection of metastasis and progression are needed. The identification of biomarkers that can be easily and regularly measured in cancer patients to detect cancer metastasis would be promising. Recently, ctDNA^59^ has been acknowledged as a promising biomarker for several types of cancer^60^,^61^,^62^,^63^,^64^. Using new PCR technologies, ctDNA can be quantitatively measured in cancer patients^65^,^66^. Dynamic changes in ctDNA levels might serve as a biomarker of early relapse in cases of surgically resected metastatic melanoma.

We also investigated the impact of heterogeneity in patient drug-metabolism rates on survival percentage. To include the effects of population heterogeneity in our mechanistic models and examine the impact of patient drug metabolism heterogeneity on survival percentage, we considered three patient subclasses with different metabolic rates (low, medium, and high). As shown in Supplementary Fig. S3, metabolic rate differences significantly affected the probability of patient survival. The patient subclass with a high rate of drug metabolism showed a lower survival probability compared to the patient subclass with a low rate of drug metabolism. This was true under both the 3 weeks on/1 week off treatment schedule (Supplementary Fig. S3A) and the daily treatment schedule (Supplementary Fig. S3C). In Supplementary Fig. S3B, D, the effects of different drug combination dosages on survival percentage in high-metabolism patients are shown. An appropriately elevated dosage of BRAF-I combined with MEK-I (2-fold of normal dosages) improved progression-free survival in cancer patients with high metabolic rates. As a strategy for personalized therapy, optimizing dosing based on patient metabolic rate might be beneficial. Furthermore, our model indicated that daily treatment of cancer patients (Supplementary Fig. S3C, D) resulted in higher survival rates versus the 3 weeks on/1 week off treatment schedule (Supplementary Fig. S3A, B). These results suggest that daily drug treatment might produce better results than therapies with drug discontinuance.

Our model predicted that combined BRAF and MEK inhibitions produced a different pattern of synergy from that created by combined BRAF and PI3K inhibition. As demonstrated in^15^, the use of a BRAF-I can induce drug-sensitive cells to secrete DIRFs that promote PI3K/AKT/mTOR pathway activation in drug-resistant cells. Thus, BRAF-I and PI3K-I co-treatment represses the actions of DIRFs on drug-resistant cells and reduces growth, dissemination and metastasis in drug-resistant cells. Therefore, at appropriate dosages, this combination therapy produced a synergistic effect, reducing the number of tumor cells (Fig. 5C,D). Conversely, because MEK is a downstream target of BRAF signaling, MEK inhibition does not necessarily strengthen BRAF inhibition. As such, BRAF-I and MEK-I co-treatment produced only weak synergistic effects at relatively low doses. This lack of cooperation led to a smaller reduction in tumor cell number (Fig. 5A,B).

Drug resistance is an obstacle often encountered during oncoprotein-targeted therapy and develops by very complex mechanisms. Tumors frequently display a substantial amount of spatial heterogeneity in both cell population composition and microenvironment factors, such as drug, oxygen and glucose concentrations^2^,^67^. Recent studies have suggested that the emergence of drug resistance is driven by the tumor microenvironment^2^. In our study, based on recent clinical and preclinical data, we modeled feedback in drug-sensitive cancer cells in response to drug treatment and interactions between heterogeneous cell populations and their microenvironments to understand drug resistance and metastasis dynamics in tumors. In future work, we aim to investigate spatial heterogeneity in tumor cells by developing an agent-based model^21^,^43^ using partial differential equations to assess spatial-temporal changes in the tumor microenvironment.

In summary, our study utilized a set of SDEs to model the dynamics of targeted therapy-induced drug resistance and metastasis. The model predicted that different patterns of synergy exist for the combination of BRAF-I and MEK-I and for the combination of BRAF-I and PI3K-I. Our study provides insight into the microenvironment-mediated mechanisms underlying drug resistance and delineates the implications associated with optimal dosing and scheduling of combination therapy for melanoma patients with BRAF mutations. It is our hope that this predictive model will facilitate cancer clinical trial design and accelerate the design of effective and robust tumor therapeutics.

## Additional Information

**How to cite this article**: Sun, X. *et al.* Mathematical Modeling of Therapy-induced Cancer Drug Resistance: Connecting Cancer Mechanisms to Population Survival Rates. *Sci. Rep.* **6**, 22498; doi: 10.1038/srep22498 (2016).

## Supplementary Material

Supplementary Information

## Figures and Tables

**Figure 1:**
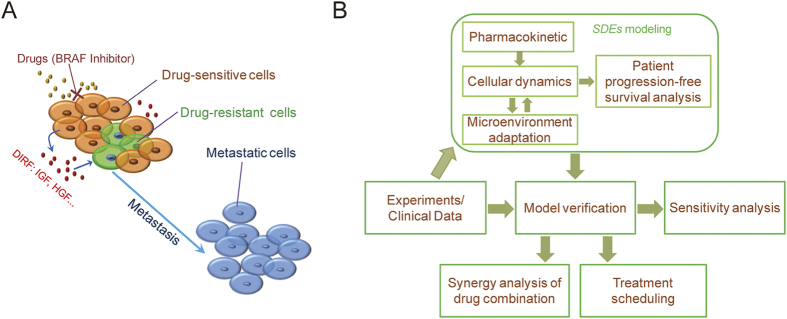
(**A**) Schematic representation of therapy-induced drug resistance and metastasis. Targeted therapy (e.g., treatment of melanoma using BRAF kinase inhibitors) is effective on drug-sensitive cells; however, a small number of pre-existing drug-resistant cancer cells are unaffected by treatment. In response to drug treatment, drug-sensitive cancer cells secrete various compounds (e.g., IGF and HGF) into the tumor microenvironment. These compounds were termed drug-induced resistance factors (DIRFs) in this study. The secreted DIRFs enhance the growth, dissemination and metastasis of cancer cells^15^. In our mathematical model, the metastatic cells refer to the new metastatic cells after the initiation of drug treatment. (**B**) A flowchart of our work. We constructed a stochastic model comprised of a set of stochastic differential equations driven by both Wiener and Poisson processes to model pharmacokinetics, DIRF secretion and cellular dynamics based on the recent experiments and clinical data. This enabled us to calculate progression-free survival *in silico*. Our model was then verified by comparing its predictions against clinical patient survival data. We used the model to quantitatively evaluate the efficiency and synergy of two combination therapies (BRAF inhibitor plus MEK inhibitor and BRAF inhibitor plus PI3K inhibitor). Furthermore, sensitivity analysis revealed several important parameters in the model that may provide implications for the design of combination therapies. In addition, we also examined cellular- and patient-level responses to different drug treatment schedules and investigated the impact of heterogeneity in drug metabolism rates on patient survival.
