# Supplementary material for: Mathematical Modeling of Therapy-induced Cancer Drug Resistance: Connecting Cancer Mechanisms to Population Survival Rates
Source: Sci Rep. 2016 Mar 1;6:22498. doi: 10.1038/srep22498 (PMC4772546; doi:10.1038/srep22498)
Supplement: Supplementary Information [file srep22498-s1.pdf]

Supplementary Information

Mathematical Modeling of Therapy-induced Cancer Drug Resistance: Connecting Cancer Mechanisms to Population Survival Rates

Xiaoqiang Sun[1,2]*, Jiguang Bao[3], Yongzhao Shao[4,5]*

1 Zhong-shan School of Medicine, Sun Yat-Sen University, Guangzhou 510089, China

2 School of Mathematical and Computational Science, Sun Yat-Sen University, Guangzhou 510000, China

3 School of Mathematical Sciences, Beijing Normal University, Beijing 100875, China

4 Department of Population Health, NYU School of Medicine, New York University, New York, NY 10016, USA

5 Interdisciplinary Melanoma Collaborative Group, NYU Cancer Institute, New York, NY 10016, USA

Text S1. Parameter calibration.

Text S2. Sensitivity analysis methods.

Figure S1. Fitting the model to the clinical data.

Figure S2. Cellular dynamics without drug treatment.

Figure S3. Impact of patient metabolic heterogeneity on patient survival.

Table S1. Values of parameters in the model.

Text S1. Parameter calibration

Biological descriptions and values of the model parameters are listed in Table S1. Most of the parameters involved in the cellular dynamics and pharmacokinetics modules were collected from previous studies [1-3], while other parameters were calibrated according to recent experimental [4, 5] and clinical data [6]. We provide details regarding the calibration of the parameter values below.

Parameters involved in DIRF secretion. Recent studies [4] (extended data, Figure 4d in Ref. [4]) have reported that the levels of some murine stroma-derived cytokines, such as HGF, IGF, MFGE8 and TREM1, were upregulated by 1.6- to 10-fold in response to treatment with Vemurafenib (a BRAF inhibitor) compared to vehicle treatment. The newly secreted DIRFs are described in equation (7) in the main text. The DIRF basal level was assumed to be 0.1. To ensure that the change in total DIRF (basal plus newly secreted DIRFs) remained within a range of 1 to 10 according to the experiments mentioned above, $\tilde{V}_{DS}$ and K_{DS1} (*i*=1,2) were set to 100 and 10, respectively, as the concentration of BRAF inhibitor changed from 0.1 to 1 in the simulation. Note that in $\tilde{V}_{DS} = V_{DS}/d_{DIRF}$, the degradation rate (d_{DIRF}) is assumed to be 0.01 per day. Thus, the maximal DIRF secretion rate from drug-sensitive cells (V_{DS}) was accordingly set to 1.

Parameters involved in drug-resistant cell growth. The experimental data in Ref. [4] also indicated that the relative number of drug-resistant cancer cells in drug-sensitive tumors increased by approximately 2- to 4-fold after 3 days of drug treatment (Vemurafenib, Crizotinib and Erlotinib).The growth rate coefficient corresponding to drug-resistant cells regulated by DIRFs is described in equation (9) in the main text. Because the minimal and maximal DIRF concentrations were set to 0.1 and 0.9, as stated in the above section, α and K_{2_DIRF} were set to 0.1 and 0.3, respectively, to ensure an approximate 2- to 4-fold change in the relative number of drug-resistant cancer cells induced by the drug treatment on day 3, in agreement with the above-mentioned experimental data. The value of K_{1_DIRF} , the DIRF-associated Michaelis constant, was set in a similar way for drug-sensitive cancer cells.

Parameters involved in metastasis. The drug-resistant cancer cell dissemination rate coefficient that was regulated by DIRFs is described by equation (10) in the main text. We set the values of α and K_{3_DIRF}

based on experimental data [4] (Figure 2D in Ref. [4]). The relative migration of drug-resistant cancer cells increased by 2- to 3-fold following BRAF inhibitor treatment compared to vehicle treatment. Therefore, to reproduce this change in our simulation, α was set to 0.3, and K_{3_DIRF} was set to 0.01. λ (intensity of Poisson process) in Equation (11) was scaled and estimated from Ref. [2].

Parameters fitted to the clinical data. The clinical data of progression-free survival percentages (Figure 1A in Ref. [6]) were used to estimate parameters including d_S (maximal inhibition rate of drug on drug-sensitive cells), K_{Tm} (Michaelis constant for maximal carrying capacity of drug-sensitive/resistant cells), M_{max} (maximal carrying capacity of new metastatic cells) and σ (diffusion coefficient in Wiener process). The clinical data include progression-free survival percentages of a patient cohort treated with daily dosing of BRAF inhibitor vemurafenib and MEK inhibitor cobimetinib administered for 21 days, followed by 7 days off. By iteratively calibration, we chose the values of the above parameters which produced good agreement with the clinical data (see **Supplementary Fig. S1**). It should be noted that another independent set of clinical data (Figure 1A in Ref. [7]) was used for validating our model predictions at patient population level, as presented in **Fig. 3** in the main text.

Text S2. Sensitivity analysis

Sensitivity analysis was used to quantitatively explore the critical parameters in the model that affected cellular dynamics and survival percentage. A sensitivity coefficient [8] for total tumor cell number with respect to parameter p_j was calculated as follows:

$$S_j^T = \frac{\int_0^T \bar{C}_T(t,\tilde{p}_j)dt - \int_0^T \bar{C}_T(t,p_j)dt}{\int_0^T \bar{C}_T(t,p_j)dt} \Bigg/ \frac{\Delta p_j}{p_j} \approx \frac{\sum_{i=1}^{L} \Delta \bar{C}_T(t_i)}{\sum_{i=1}^{L} \bar{C}_T(t_i)} \Bigg/ \frac{\Delta p_j}{p_j} \qquad \textbf{(S1)}$$

where $\{t_i, i=1,\cdots L\}$ is an equal partition of $[0,T]$, with L =360 and T =360 days in the simulation. $\bar{C}(t,p_j)$ and $\bar{C}(t,\tilde{p}_j)$ represent the median total tumor cell numbers with parameter p_j and varied parameter $\tilde{p}_j$ at time t respectively. $\Delta\bar{C}_T(t_i) = \bar{C}_T(t_i,\tilde{p}_j) - \bar{C}_T(t_i,p_j)$ is the change of median total tumor cell number with the parameter perturbation $\Delta p_j = \tilde{p}_j - p_j$ at time t_i (day). Then, we obtained the relative changes for the area under curve of total tumor cell number with respect to the examined parameters.

The sensitivity coefficient of the survival percentage with respect to parameter p_j was also calculated according to the following formula:

$$S_j^{SP} = \frac{\int_0^T SP(t,\tilde{p}_j)dt - \int_0^T SP(t,p_j)dt}{\int_0^T SP(t,p_j)dt} \Bigg/ \frac{\Delta p_j}{p_j} \approx \frac{\sum_{i=1}^{L} \Delta SP(t_i)}{\sum_{i=1}^{L} SP_T(t_i)} \Bigg/ \frac{\Delta p_j}{p_j} \qquad \textbf{(S2)}$$

where $SP(t,p_j)$ and $SP(t,\tilde{p}_j)$ represent survival percentages with parameter p_j and varied parameter $\tilde{p}_j$ at time t respectively. $\Delta SP(t_i) = SP(t_i,\tilde{p}_j) - SP(t_i,p_j)$ is the change of survival percentage with the parameter perturbation $\Delta p_j = \tilde{p}_j - p_j$ at time t_i (day). Then, we obtained the relative changes for the area under curve of survival percentage with respect to the examined parameters.

Single-parameter sensitivity analysis

Each parameter was increased by 50% from its estimated value and we then obtained the relative changes in area under curve of total tumor cell number and area under survival curve, as defined by equations (S1-S2). The computations were repeated 20 times, and the mean value and standard deviation of the sensitivity coefficients were calculated.

Two-parameter sensitivity analysis

To assess the combinatorial effects of parameters involved in the model, we performed a two-parameter sensitivity analysis. The values of each pair of two different parameters were increased by 50% from their original values simultaneously. All other parameters remained at their base values. Taking into account the stochasticity of the model, the computations were repeated 20 times, and the mean value of the sensitivity coefficients was calculated.

Figure S1. Fitting the model to the clinical data

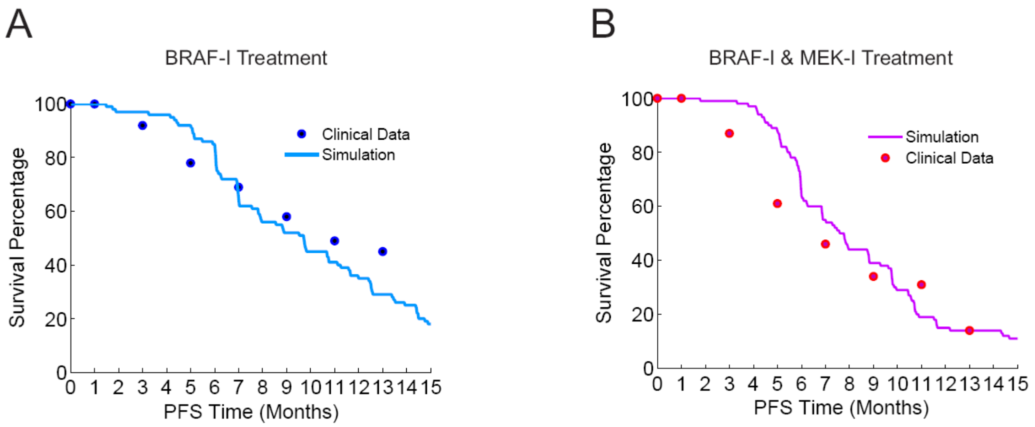

Comparisons between the simulations and the clinical data [6] of patient survival percentages with (**A**) BRAF-I treatment and (**B**) BRAF-I&MEK-I treatment respectively. The mean squared relative error (MSRE) is 0.1910.

Figure S2*. *Cellular dynamics without drug treatment

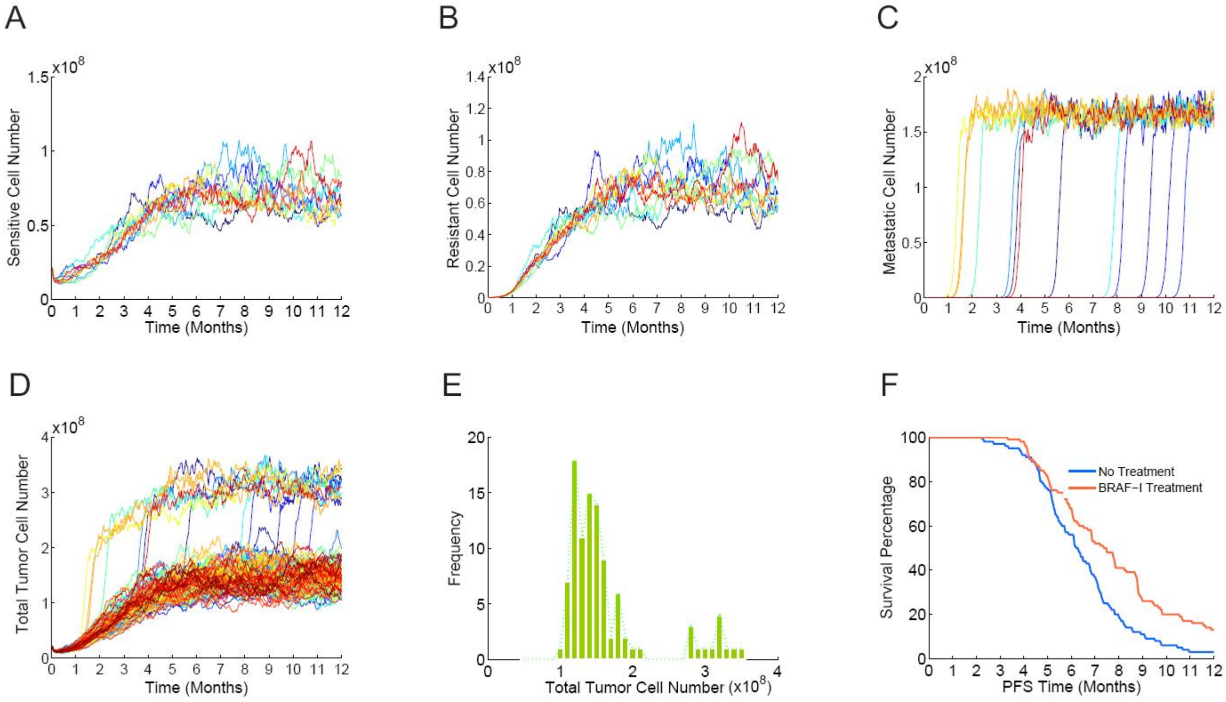

Time courses of 10 samples showing relative numbers of (**A**) drug-sensitive cancer cells, (**B**) drug-resistant cancer cells, and 100 samples showing relative numbers of (**C**) new metastatic cells and (**D**) total tumor cells. (**E**) Distribution of total tumor cell number at 450 days. (**F**) Progression-free survival percentages of patients with BRAF-I treatment (red line) and without treatment (blue line).

Figure S3. Impact of patient metabolic heterogeneity on patient survival

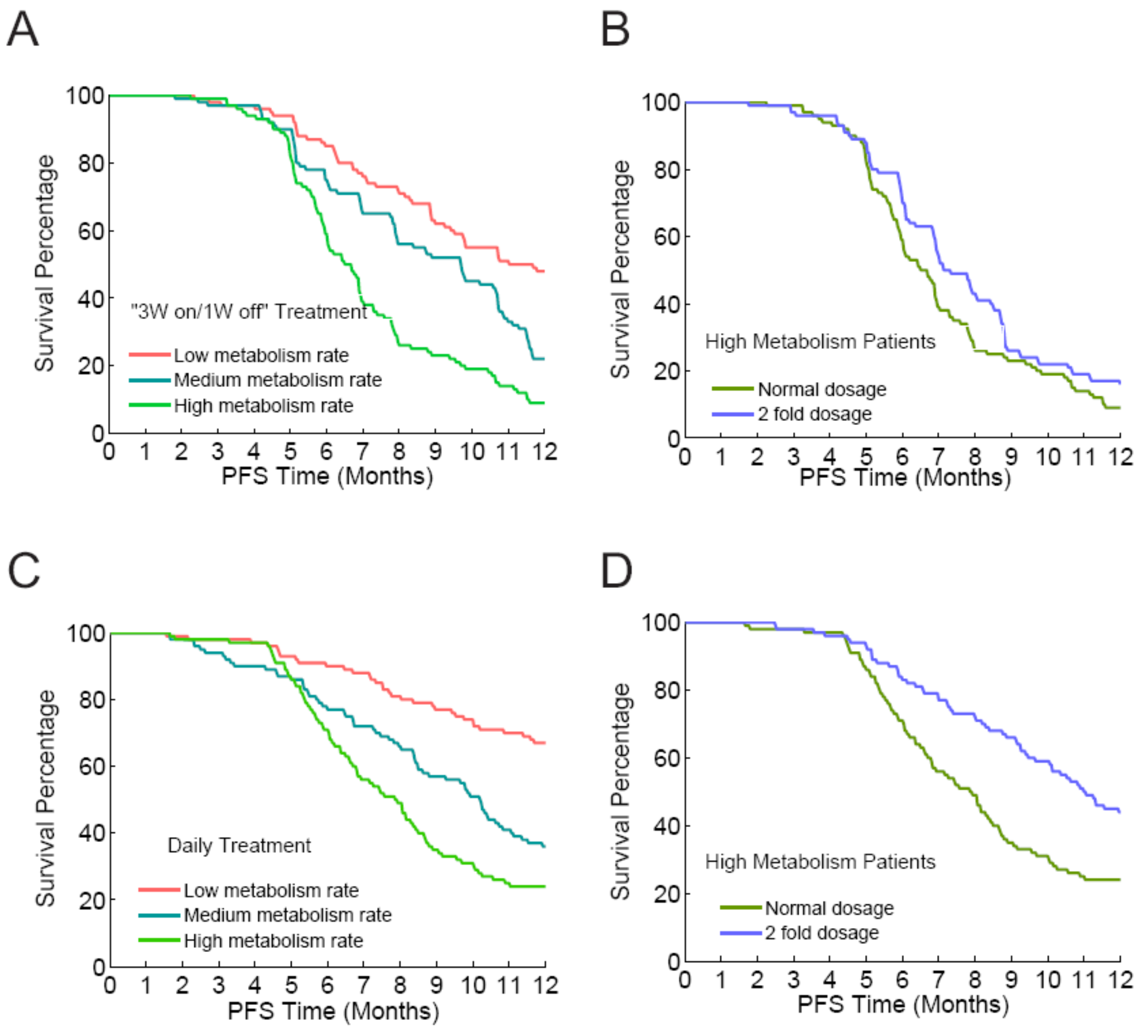

(**A**) Impact of heterogeneity in patient drug metabolism rates (low, medium and high) on survival percentage under a 3 weeks on/1 week off treatment schedule. (**B**) Survival percentage of patients with high metabolisms under a 3 weeks on/1 week off treatment schedule using different dosages of BRAF and MEK inhibitors (BRAF-I and MEK-I) in combination. (**C**) Impact of heterogeneity in drug metabolism rates (low, medium and high) on survival percentage under a daily treatment schedule. (**D**) Survival percentage of patients with high metabolisms under a daily treatment schedule using different dosages of BRAF-I and MEK-I in combination.

Table S1. Values of parameters in the model. Each parameter is listed with its symbol, value, biological description and reference.

Symbol	Value	Unit	Description	Reference
r_S	0.192	Day^{-1}	Growth rate coefficient of drug-sensitive cells	[1]
r_R	0.192	Day^{-1}	Growth rate coefficient of drug-resistant cells	[1]
r_M	0.192	Day^{-1}	Growth rate coefficient of metastatic cells	[1]
r_K	0.01	Day^{-1}	Growth rate coefficient of angiogenic cells	Scaled from [1]
d_K	0.00873	Day^{-1}	Death rate coefficient of angiogenic cells	Scaled from [1]
q_M	$1*10^{-4}$	_	Dissemination rate of metastatic cells	Scaled from [2]
u	$4*10^{-5}$	_	Mutation rate of sensitive cells to resistant cells	[9]
d_S	0.366	Day^{-1}	Maximal inhibition rate of drug on drug-sensitive cells	Calibrated to [6]
d_{DIRF}	0.01	Day^{-1}	DIRF degradation rate	Estimated from [4]
V_{DS}	1	Day^{-1}	Maximal DIRF secretion rate from drug-sensitive cells	Estimated from [4]
K_{DS}	10	_	Michaelis constant of DIRF secretion	Estimated from [4]
λ	0.02	_	Intensity of Poisson process	Estimated from [2]
K_λ	0.2	_	Michaelis constant of DIRF secretion for Poisson intensity	Estimated from [2]
d_{drug}	0.04	Day^{-1}	Elimination rate constant of drug	Scaled form [3]
K_{BRAFi}	0.5	–	Michaelis constant of BRAF inhibitor	Assumed
K_{MEKi}	0.1	–	Michaelis constant of MEK inhibitor	Assumed
K_{PI3Ki}	0.1	–	Michaelis constant of PI3K inhibitor	Assumed
K_{1_DIRF}	0.3	–	Michaelis constant of DIRF for r_S	Estimated from [4]
K_{2_DIRF}	0.3	–	Michaelis constant of DIRF for r_R	Estimated from [4]

K_{3_DIRF}	0.1	–	Michaelis constant of DIRF for q_M	Estimated from [4]
α	0.01	_	Regulatory coefficient	Estimated from [4]
C_{Th}	1.6	10^8	Critical size of total tumor cells detected as progression	Assumed
K_{Tm}	10	–	Michaelis constants for maximal carrying capacity of drug-sensitive/resistant cells	Calibrated to [6]
$M_{\max}$	1.67	10^8	Maximal carrying capacity of new metastatic cells	Calibrated to [6]
σ	0.02	_	Diffusion rate	Calibrated to [6]
C_{S0}	0.2	10^8	Initial value of the density of drug-sensitive cells	Assumed
C_{R0}	0.001	10^8	Initial value of the density of drug-resistant cells	Assumed
C_{K0}	0.1	10^8	Initial value of the density of angiogenic cells	Assumed
C_{M0}	0	10^8	Initial value of the density of new metastatic cells	Assumed

Remarks: "_" in the above Table S1 represents dimensionless unit.

References

1. Hahnfeldt, P., et al., *Tumor development under angiogenic signaling a dynamical theory of tumor growth, treatment response, and postvascular dormancy.* Cancer Res, 1999. **59**(19): p. 4770-4775.
2. Haeno, H., et al., *Computational modeling of pancreatic cancer reveals kinetics of metastasis suggesting optimum treatment strategies.* Cell, 2012. **148**(1): p. 362-375.
3. Leander, J., et al., *Mixed effects modeling using stochastic differential equations: illustrated by pharmacokinetic data of nicotinic acid in obese Zucker rats.* AAPS J, 2015. **17**(3): p. 586-596.
4. Obenauf, A.C., et al., *Therapy-induced tumour secretomes promote resistance and tumour progression.* Nature, 2015.
5. Straussman, R., et al., *Tumour micro-environment elicits innate resistance to RAF inhibitors through HGF secretion.* Nature, 2012. **487**(7408): p. 500-504.
6. Larkin, J., et al., *Combined vemurafenib and cobimetinib in BRAF-mutated melanoma.* N Engl J Med, 2014. **371**(20): p. 1867-1876.
7. Flaherty, K.T., et al., *Combined BRAF and MEK inhibition in melanoma with BRAF V600 mutations.* N Engl J Med, 2012. **367**(18): p. 1694-1703.
8. Sun, X., et al., *Systems Modeling of Anti-apoptotic Pathways in Prostate Cancer: Psychological Stress Triggers a Synergism Pattern Switch in Drug Combination Therapy.* PLoS Comput Biol, 2013. **9**(12): p. e1003358.
9. Michor, F., et al., *Dynamics of chronic myeloid leukaemia.* Nature, 2005. **435**(7046): p. 1267-1270.
